# Supplementary material for: Investigation of Pretreatment of Textile Wastewater for Membrane Processes and Reuse for Washing Dyeing Machines
Source: Membranes (Basel). 2022 Apr 21;12(5):449. doi: 10.3390/membranes12050449 (PMC9144798; doi:10.3390/membranes12050449)
Supplement: Supplementary file 1 [file membranes-12-00449-s001.zip › membranes-1688612-supplementary.pdf]

*Supplementary Material*

# Investigation of Pretreatment of Textile Wastewater for Membrane Processes and Reuse for Washing Dyeing Machines

**Iva Ćurić * and Davor Dolar**

Department of Physical chemistry, Faculty of Chemical Engineering and Technology, University of Zagreb, Marulićev Trg 19, HR-10000 Zagreb, Croatia; dolar@fkit.hr
* Correspondence: icuric@fkit.hr; Tel.: +385-1-4597-210

**Table S1.** Results of jar test experiment with $FeCl_3$.

| *c*($FeCl_3$) / mM | pH | Turbidity / NTU | pH2 | $SAC_{436\ nm}$ / $m^{-1}$ | $SAC_{525\ nm}$ / $m^{-1}$ | $SAC_{620\ nm}$ / $m^{-1}$ | TOC / mg $L^{-1}$ |
|---|---|---|---|---|---|---|---|
| 2.42 | 3.52 | 54.6 | 2.96 | 202 | 111 | 78 | 328.2 |
| 2.76 | 3.52 | 20.1 | 2.90 | 146 | 83 | 62 | 327.6 |
| 3.10 | 3.52 | 31.9 | 2.93 | 198 | 126 | 90 | 314.7 |
| 3.45 | 3.52 | 30.1 | 2.88 | 173 | 110 | 79 | 281.9 |
| 3.79 | 3.52 | 27.9 | 2.84 | 143 | 85 | 60 | 304.2 |
| 4.48 | 3.52 | 36.7 | 2.86 | 173 | 103 | 72 | 308.4 |
| 2.42 | 6.06 | 75.8 | 5.66 | 255 | 133 | 81 | 312.5 |
| 2.76 | 6.06 | 36.6 | 5.52 | 151 | 76 | 48 | 287.1 |
| 3.10 | 6.06 | 26.1 | 5.45 | 129 | 66 | 43 | 298.5 |
| 3.45 | 6.06 | 13.4 | 5.25 | 93 | 45 | 31 | 277.0 |
| 3.79 | 6.06 | 15.9 | 4.88 | 104 | 52 | 37 | 250.0 |
| 4.48 | 6.06 | 13.8 | 4.71 | 98 | 50 | 36 | 240.3 |
| 2.42 | 7.49 | 202 | 7.14 | 1264 | 741 | 484 | 382.1 |
| 2.76 | 7.49 | 248 | 6.96 | 752 | 425 | 271 | 331.6 |
| 3.10 | 7.49 | 192 | 6.89 | 727 | 403 | 250 | 327.9 |
| 3.45 | 7.49 | 149 | 6.79 | 657 | 367 | 229 | 309.2 |
| 3.79 | 7.49 | 141. | 6.57 | 438 | 261 | 170 | 327.8 |
| 4.48 | 7.49 | 94.7 | 6.43 | 232 | 128 | 81 | 313.0 |
| 2.42 | 8.66 | 43.9 | 7.19 | 1128 | 636 | 402 | 313.9 |
| 2.76 | 8.66 | 212 | 7.06 | 944 | 532 | 333 | 299.2 |
| 3.10 | 8.66 | 196 | 6.92 | 828 | 478 | 303 | 291.7 |
| 3.45 | 8.66 | 165 | 6.88 | 742 | 425 | 268 | 285.0 |
| 3.79 | 8.66 | 111 | 6.78 | 480 | 276 | 174 | 281.8 |
| 4.48 | 8.66 | 65.6 | 6.60 | 310 | 171 | 104 | 268.3 |

*The ANOVA for the obtained models (M-TOC, M-TUR, M-RpH, and M-COLOR) shows that they are significant. In this order, the $R^2$ of M-TOC, M-TUR, M-RpH, and M-COLOR were 0.8882, 0.5242, 0.9797 and 0.8312, respectively.

**Table S2.** Results of jar test experiment with $FeCl_3$/MagnaFloc LT25.

| *c*(MagnaFloc LT25) / mg $L^{-1}$ | pH | Turbidity / NTU | pH2 | $SAC_{436\ nm}$ / $m^{-1}$ | $SAC_{525\ nm}$ / $m^{-1}$ | $SAC_{620\ nm}$ / $m^{-1}$ | TOC / mg $L^{-1}$ |
|---|---|---|---|---|---|---|---|
| 0.10 | 5.95 | 12.2 | 4.91 | 74 | 42 | 34 | 269.6 |
| 0.20 | 5.95 | 6.4 | 4.78 | 49 | 29 | 38 | 265.4 |
| 0.30 | 5.95 | 10.9 | 4.82 | 53 | 35 | 41 | 270.6 |
| 0.50 | 5.95 | 9.4 | 4.91 | 59 | 40 | 41 | 272.7 |
| 0.75 | 5.95 | 10.7 | 4.89 | 71 | 40 | 41 | 281.6 |
| 1 | 5.95 | 13.1 | 4.99 | 75 | 37 | 36 | 262.7 |

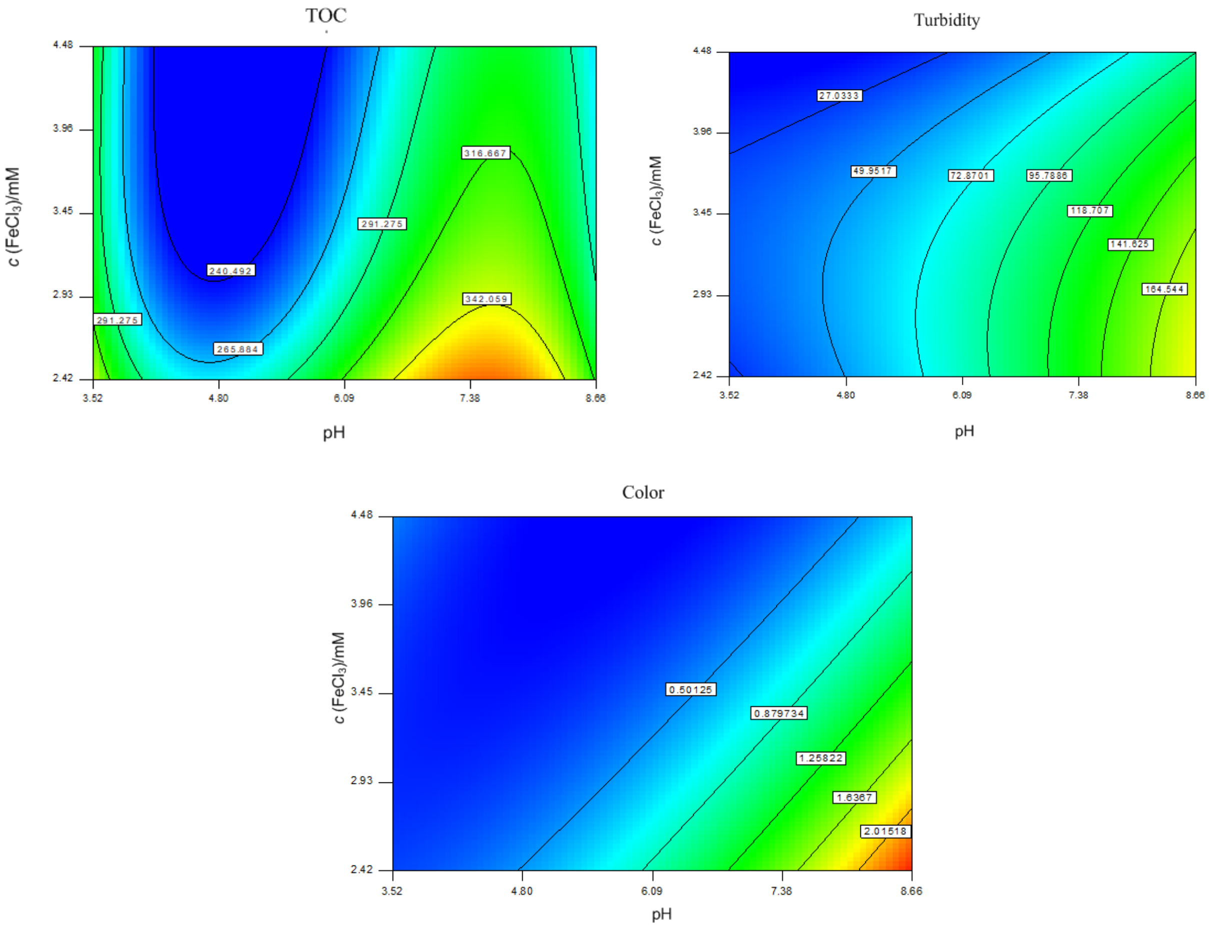


**Figure S1.** Response surfaces of residual TOC, turbidity, and color for coagulation with $FeCl_3$.
